# Prevalence and predictors of short stature in children aged 3–18 years in Hainan Province, China: a cross-sectional study

**DOI:** 10.3389/fped.2025.1522060

**Published:** 2025-01-20

**Authors:** Mi Yan, Yingying Qin, Hongai Li, Chuican Huang, Haidan Li, Li Liu, Yuhua Cai, Lichun Fan, Wei Xiang, Xiaoyan Huang

**Affiliations:** ^1^ Hainan Women and Children's Medical Center, Hainan Medical University, Hainan Academy of Medical Sciences, Haikou, China; ^2^ School of Pediatrics, Hainan Medical University, Haikou, China

**Keywords:** short stature, child, cross-sectional study, nomogram, risk factors

## Abstract

**Background:**

Short stature is a global health-related problem and remains to be evaluated in children in Hainan Province. The present study was conducted to investigate the associated factors with short-stature in children.

**Methods:**

This cross-sectional study was conducted using a staged, cluster random sampling method. A total of 26,189 children aged 3–18 years, originating from 18 cities and counties in Hainan Province, to determine the prevalence of short stature. Additionally, these children were selected for a thorough analysis of associated factors.

**Results:**

The crude prevalence of short stature was 2.88%, and the standard prevalence was (after adding weights) 3.01%. Children's short stature was significantly related to seven factors: area, birth weight, body mass index z score (BAZ), maternal education, family income (RMB per year), frequency of bean intake and frequency of egg intake. A nomogram model of factors associated with short stature was constructed. The area under the curve (AUC) of the receiver operating characteristic (ROC) curve was 0.698 (95% CI: 0.679–0.716).

**Conclusions:**

Our findings demonstrated that birth weight, BAZ, dietary habits, and family-related factors were strongly correlated with short stature in children in Hainan Province.

## 1 Introduction

A child's height-for-age z score (HAZ) tells us how many z scores (standard deviations) (SD) away from the mean of a reference population, considering both age and sex. If the child's HAZ is below 2 SD then this child is considered to have stunting/short stature (i.e., low height compared to a healthy reference child of the same age and sex) according to the WHO (World Health Organization) growth reference (1, 2). It is estimated that there are more than 150 million children with short stature worldwide (3). In addition, the prevalence of short stature is highly variable across different countries and areas, possibly due to different ethnicities and geographic locations (4). The prevalence of short stature in foreign children is approximately 1.82%–15% (2, 5, 6). In 2019, Yanhui Dong and colleagues conducted a survey among adolescents aged 7–18 across 29 provinces in China, revealing an average prevalence rate of short stature among Chinese children and adolescents to be 2.4%, based on WHO standards (7). Shaojun Huang and his team investigated the prevalence of short stature in 12,504 children aged 6–14 in Shenzhen, China, finding a prevalence rate of 4.3% in the areas (8). A 2020 study surveyed 9,501 children aged 3–6 in Beijing, China, and discovered a prevalence rate of 4.3% in the area (9). Meanwhile, the prevalence rate among children aged 6–16 in Jining, Shandong Province, China, was reported to be 3.16% (10). (Note: The prevalence rates reported for Shenzhen, Beijing, and Shandong in China are based on Chinese standards). Currently, some scholars are researching short stature (11, 12).

Short stature is one of the most common disorders in paediatric endocrinology (13, 14). The adverse effects of short stature on children and adults should not be overlooked. It has been reported that children with short stature may have a poorer quality of life. For example, children with short stature are subject to peer ridicule and increased psychosocial stress, and may exert an increased burden of care on their parents (15). In addition, children with short stature have fewer opportunities for higher education and employment and are likely to have lower incomes in adulthood (16, 17). Furthermore, short stature is associated with diseases of the cardiovascular system, such as diabetes and coronary heart disease (18–20). Therefore, obtaining up-to-date data on short stature and clarifying the factors that influence height are of great importance and clinical value for public health. Early detection and intervention can not only increase height but can also reduce psychosocial disability (21). The consensus is that height is multifactorial, including genetic, environmental, nutritional, social, and other factors (22–24). Notably, from the end of the 1990s to the present, many researchers have shown that parental genetics influence the height of their children (25–27). There is a strong link between parental height and short stature in offspring (28–30). However, the results are often inconsistent for other nongenetic factors. For instance, a study in the Gaza Strip, Palestine, showed no significant correlation between parental education and the short stature of children (31). In contrast, another study in Saudi Arabia reported a significant correlation between parental education and the short stature of children (5). This discrepancy may be due to other factors, such as the sample population and methodology. To further investigate the impact of nongenetic factors on children's short stature in children in Hainan Province, we conducted a large-scale cross-sectional survey of children between the ages of 3 and 18 years in Hainan Province. This study had two objectives. First, we aimed to investigate the prevalence of short stature in children in Hainan Province. Second, we aimed to identify nongenetic predictors of short stature in children and to provide early and timely intervention for children's height. In this work, we endeavoured to develop a predictive model for assessing the risk of short stature in children in Hainan Province. However, there are no studies related to short stature among children in Hainan Province. In this study, the predictors of short stature in children in Hainan Province are analysed for the first time with the aim of preventing short stature in adults.

## 2 Methods

### 2.1 Study population

This study used data from the Hainan Provincial Major Science and Technology Plan Project, titled “Research on Growth and Development Monitoring and Influencing Factors of Children (aged 0–18) in Hainan Province, ZDKJ2019010.” We selected 18 cities and counties in Hainan Province as our sampling areas. Taking into account the feasibility of the research survey and the representativeness of the samples, we employed a continuous recruitment method through township and community healthcare units. We used a staged, cluster random sampling method. (specific methods of sampling can be found in Supplementary Appendix S1). Finally, 26,189 children were selected to participate in the cross-sectional survey.

Inclusion and exclusion criteria: The inclusion criterion was children aged 3–18 years whose parents agreed to participate in the research and understood the questionnaire.

The exclusion criteria were as follows: (1) children who are permanent residents of Hainan Province or have migrated from other places but have lived in Hainan for more than two-thirds of their age; (2) those with the following diseases: heart disease (murmur of grade II or above); chronic nephritis, tuberculosis, migratory hepatitis, endemic diseases, chronic bronchitis, and asthma; endocrine diseases; neurological disorders; rickets of a moderate level or above or other deformities affecting the development of the physical body; or disability of the limbs, as well as those who had recovered from the initial stage of an acute disease (e.g., pneumonia and dysentery) less than a month prior and those who have had a fever for more than 7 days or diarrhoea for more than 5 days in the last two weeks; (3) there are missing or incomplete responses in the questionnaire.

### 2.2 Sample weighting

A total of 26,189 children aged 3–18 years, including 12,996 boys and 13,193 girls, were surveyed in 18 cities and counties in Hainan Province in this cross-sectional study. There was a significant difference between this sample and the whole Hainan Province population sample in 2020 in terms of the characteristics of the population's demographic composition. The statistical description of this cross-section was weighted for each study population, and then statistical analysis was completed. After the cross-sectional population was weighted, the population composition ratio was closer to the total population composition ratio of the population aged 3–18 years in 2020.

### 2.3 Sample size estimation

Previous research showed that the prevalence of short stature in Chinese individuals was 3% (32). The sample size of 17,823 was calculated according to the following sample content estimation formula (taking *α* = 0.05, *δ* = 0.0025, *P* = 0.03, *N* = 5,000,000). Estimating that 20% of the data may be missing, the final sample size was set at *N* = 26,189.

$$N=\frac{Z_{\alpha /2}^{2}P(1-P)N}{\delta^{2}(N-1)+Z_{\alpha /2}^{2}P(1-P)}$$

### 2.4 Questionnaire collection

We designed an electronic questionnaire. The questionnaires were completed by the child's primary caregiver, who was guided by uniformly trained investigators. The questionnaires were entered into the “Research System on Growth and Development Monitoring and Influencing Factors of Infants and Young Children Aged 0–18 in Hainan Province” system developed by the research group (Certificate No.: Softwriting Registration No. 10456369, URL: https://dl.hnwcmc.com/#/login).

### 2.5 Quality control

The investigators were trained before the official start of the questionnaire survey. The quality of work in the field was checked at multiple levels during the survey. Each questionnaire collected was subjected to a preliminary, intermediate and final review. The reviewed questionnaires were entered into the computer using the second entry (double entry) method in accordance with a uniform entry format. The data was verified using the computer and corrected by checking with the original card when errors were found.

### 2.6 Content of the questionnaire

The questionnaire included questions about the children's height, weight, gender, area, birth weight, paternal/maternal education, family income (RMB per year), weekly intake frequencies of staple foods, fruits, beans, meat, and eggs, weekly intake frequencies of puffed snacks, fried snacks, cakes/biscuits snacks, drinks, sweets/chocolate snacks, daily outdoor activity time, and daily electronic screen time.

In addition, the measurement of height and weight can be found in Supplementary Appendix S2.

### 2.7 Definitions and criteria for classification

Education level is divided into five categories: primary school and below, middle school, high school/secondary school, undergraduate/graduate college, and graduate school and above. Family income (RMB per year) level is categorized into five brackets: 0–30,000, 30,000–50,000, 50,000–100,000, 100,000–300,000 and ≥300,000.

Height-for-age z score (HAZ): The difference between the measured height of a child and the mean height of a reference child of the same age and gender and the standard deviation (SD) of the reference child's height is the height-for-age z score.

A child's HAZ tells us how many z scores (SD) away from the mean of a reference population, considering both age and gender. If the child's HAZ is below 2 standard deviations then this child is considered to have stunting (i.e., low height compared to a healthy reference child of the same age and gender) (1).

Body Mass Index for Age Z score (BAZ) is a statistical measure that compares a child's body mass index (BMI) to the BMI of a reference population of children of the same age and gender. Like HAZ, BAZ allows for the assessment of a child's nutritional status relative to a healthy standard. According to WHO standards, for children aged 0–5 years, a BAZ below −2 SDs indicates thinness, a BAZ within the range of −2 to +2 SDs indicates normal weight, a BAZ above +2 SDs but below +3 SDs indicates overweight, and a BAZ above +3 SDs indicates obesity. For children aged 5–18 years, the cut-offs are slightly different, with a BAZ below −2 SDs indicating thinness, a BAZ within the range of −2 SDs to +1 SD indicating normal weight, a BAZ above +1 SD but below +2 SDs indicating overweight, and a BAZ above +2 SDs indicating obesity (1, 33).

BMI, HAZ and BAZ were calculated using the WHO-recommended Anthro and AnthroPlus software.

Lifestyle factors considered in the study included dietary habits, time spent on electronic screens, and time spent engaging in outdoor activities. Dietary habits included weekly intake frequencies of beans, meat, eggs, staples, fruits, puffed snacks, fried snacks, cake/biscuit snacks, drinks, and sweet/chocolate snacks. Weekly intake rates were categorised as none, occasional (1–2 times), often (3–5 times), or daily. Daily electronic screen time and outdoor activity time were classified into 3 categories: <1 h, 1–2 h and >2 h. The textures of the staple foods were divided into four categories: gruel, thick porridge, thin rice, and dry rice. The criteria for categorisation of urban and rural areas can be found in Supplementary Appendix S3.

### 2.8 Statistical analysis

The *χ*^2^ test was used to evaluate the prevalence of short stature among children of different genders and ages in Hainan Province. In the analysis of influencing factors, *χ*^2^ test and *t* test were used to calculate the differences between the short stature and normal groups in terms of basic information, family-related factors and lifestyle-related factors. Categorical variables were expressed as counts (%), and continuous variables were expressed as means ± standard deviation ($\bar{x}\pm s$). Subsequently, univariate and multivariate logistic regression analyses were employed to identify significant factors contributing to short stature. The 95% confidence intervals (CIs) and *P* values were recorded. And, a Random Forest plot was constructed to visualize the factors that may be associated with short stature. Finally, a risk prediction nomogram model for childhood short stature was constructed. The area under the curve (AUC) of the receiver operating characteristic (ROC) curve was used to evaluate the discriminatory power of the predictive model.

**Figure 1:**
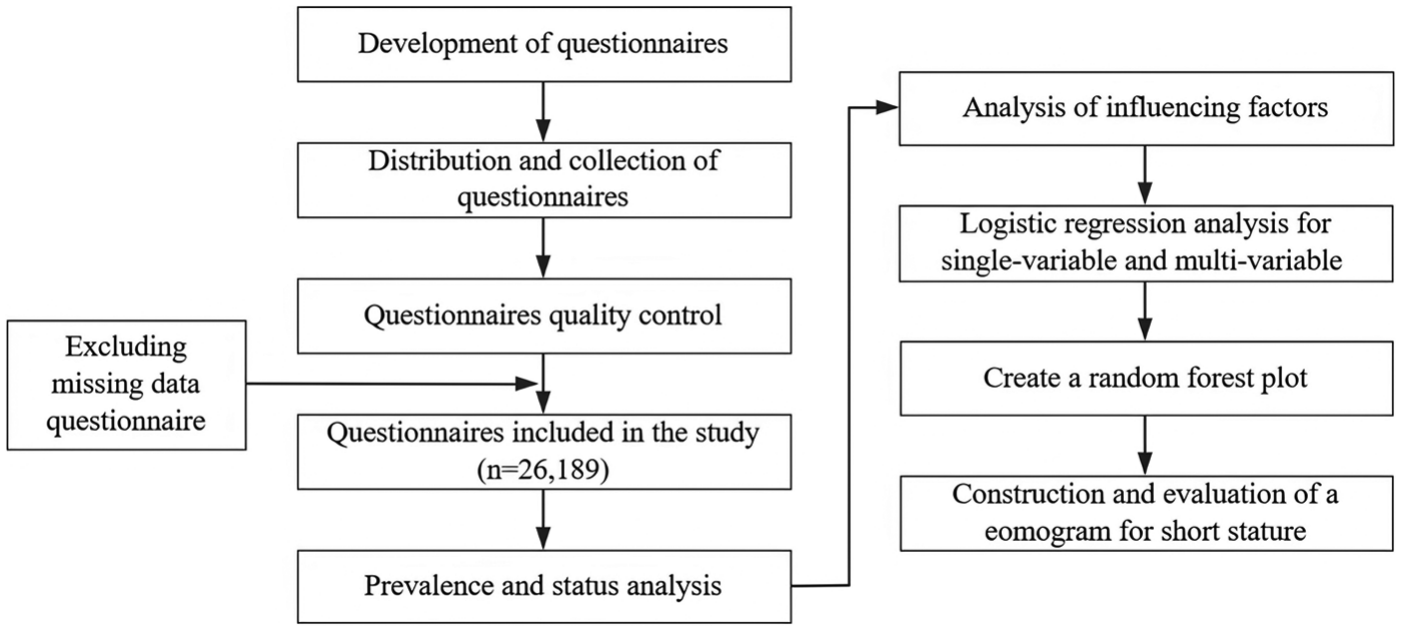
Flowchart of the data screening process.

Statistical analyses were completed using SPSS software (version 26.0) and R (version 4.3.3). A *P* value < 0.05 was considered to indicate statistical significance. Figure 1 shows the flow chart for this study. The variable assignment table can be found in Supplementary Appendix S4.

## 3 Results

### 3.1 Baseline characteristics

In this study, of the 26,189 children included, 753 had short stature, with a crude prevalence of 2.88% and a standardised prevalence of 3.01%. The underlying prevalence is shown in Table 1. The standard prevalence of short stature was 2.90% in boys and 3.10% in girls, with no statistically significant difference between the male and female groups (*p* > 0.05).

**Table 1:** Prevalence of short stature in children of different genders and ages in Hainan Province, China, 2022 (*n* = 26,189).

Characteristics	Frequency (n)	Crude prevalence (%)	Standardised prevalence (%)
Age (years)			
3	19	3.06	2.91
4	39	5.39	4.66
5	20	2.77	2.32
6	53	2.05	2.33
7	86	2.66	2.64
8	80	2.68	2.67
9	92	3.11	3.00
10	78	2.62	2.62
11	64	2.14	2.14
12	36	2.67	2.55
13	28	2.65	2.88
14	26	2.54	3.02
15	35	3.23	3.12
16	44	4.61	4.52
17	53	5.68	5.19
Total	753	2.88	3.01
Gender			
Females	389	2.95	3.10
Males	389	2.80	2.90
Area			
Rural	604	3.59	3.76
Urban	149	1.59	1.71

Crude prevalence was calculated as the prevalence of the 26,189 children who were included; standardised prevalence was calculated by adding weights.

### 3.2 Identification of significant predictors

A total of 26,189 questionnaires were included in the analysis of relevant factors. Table 2 shows that children and adolescents in the short stature group had significantly lower parental education and annual family income than those in the normal group (*P* < 0.001), and a significantly greater percentage of short group children lived in rural areas (*P* < 0.001). In terms of dietary habits, the frequency of weekly intake of beans, meat, eggs and fruits was significantly greater in the normal group than in the short stature group (*P* < 0.001). In terms of the texture factor of staple foods, the percentage of dry rice consumed was significantly greater in the normal group of children than in the short stature group (*P* < 0.001), but there were no statistically significant differences in puffed snack intake, fried snack intake, biscuits-cakes intake, drinks intake, sweets-chocolate intake, electronic screens, outdoor activities (*P* > 0.05) (Supplementary Appendix S5). In terms of personal factors, birth weight <2,500 g, gestational age < 37 weeks and thinness were significantly greater in the short stature group than in the normal group (*P* < 0.001). All significant factors were subsequently included in logistic regression analyses.

**Table 2:** Baseline characteristics of the study children (*n* = 26,189).

Variables	Children with short stature (*n* = 753)	Children with normal height (*n* = 25,436)	*χ*2/*t*	*P* value
Basic information				
Gender (%)			0.511	0.475
Females	389 (51.66)	12,804 (50.34)		
Males	364 (48.34)	12,632 (49.66)		
Age (years)	9.93 ± 3.79	9.58 ± 3.35	25.677	0.006
Area (%)			85.686	<0.001
Rural	604 (80.21)	16,231 (63.81)		
Urban	149 (19.79)	9,205 (36.19)		
Gestational age (weeks) (%)			18.892	<0.001
37–42	643 (85.39)	22,840 (89.79)		
<37	85 (11.29)	1,821 (7.16)		
≥42	25 (3.32)	775 (3.05)		
Birth weight (%)			88.851	<0.001
2,500–4,000 g	640 (84.99)	22,948 (90.22)		
<2,500 g	93 (12.35)	1,248 (4.91)		
≥4,000 g	20 (2.66)	1,240 (4.87)		
BAZ (%)			124.163	<0.001
Normal	620 (82.34)	19,811 (77.89)		
Thin	84 (11.16)	1,138 (4.47)		
Overweight/Obesity	49 (6.50)	4,487 (17.64)		
Family-related factors				
Maternal education (%)			104.231	<0.001
Elementary school and below	139 (18.46)	2,526 (9.93)		
Middle school	462 (65.35)	14,097 (55.42)		
High school/Secondary school	98 (13.02)	5,006 (19.68)		
Undergraduate/University college	54 (7.17)	3,737 (14.69)		
Graduate school and above	0 (0.00)	70 (0.28)		
Paternal education (%)			79.425	<0.001
Elementary school and below	79 (10.49)	1,653 (6.50)		
Middle school	468 (62.15)	13,063 (51.36)		
High school/Secondary school	139 (18.46)	6,029 (23.70)		
Undergraduate/University college	66 (8.77)	4,584 (18.02)		
Graduate school and above	1 (0.13)	107 (0.42)		
Family income (RMB per year) (%)			109.450	<0.001
0–30,000	514 (68.26)	13,037 (51.25)		
30,000–50,000	154 (20.45)	5,613 (22.07)		
50,000–1,00,000	63 (8.37)	4,351 (17.11)		
1,00,000–3,00,000	18 (2.39)	2,083 (8.19)		
≥3,00,000	4 (0.50)	352 (1.38)		
Lifestyle-related factors				
Staple intake (%)			10.491	0.015
None	97 (12.88)	2,474 (9.73)		
Occasional	104 (13.81)	3,214 (12.63)		
Often	56 (7.44)	1,823 (7.17)		
Every day	496 (65.87)	17,925 (70.47)		
Fruit intake (%)			22.277	<0.001
None	117 (15.54)	2,891 (11.36)		
Occasional	145 (19.25)	3,909 (15.37)		
Often	117 (15.54)	3,858 (15.17)		
Every day	374 (49.67)	14,778 (58.10)		
Beans intake (%)			38.256	<0.001
None	267 (35.46)	6,530 (25.67)		
Occasional	313 (41.57)	11,574 (45.50)		
Often	110 (14.61)	4,722 (18.56)		
Every day	63 (8.36)	2,610 (10.26)		
Meats intake (%)			23.358	<0.001
None	111 (14.74)	2,753 (10.82)		
Occasional	164 (21.78)	4,689 (18.44)		
Often	146 (19.39)	4,800 (18.87)		
Every day	332 (44.09)	13,194 (51.87)		
Eggs intake (%)			43.990	<0.001
None	161 (21.38)	3,550 (13.96)		
Occasional	291 (38.65)	9,557 (37.57)		
Often	186 (24.70)	6,800 (26.73)		
Every day	115 (15.27)	5,529 (21.74)		

BMI z: BMI z: BMI z score. The data are expressed as the mean (interquartile ranges) or counts (percentages). The *P* value was calculated using *t* test or chi-squared test. A *p* value < 0.05 indicated statistical significance. Weekly intake frequency was classified as none, often (3–5 times), occasional (1–2 times) or daily.

**Figure 2:**
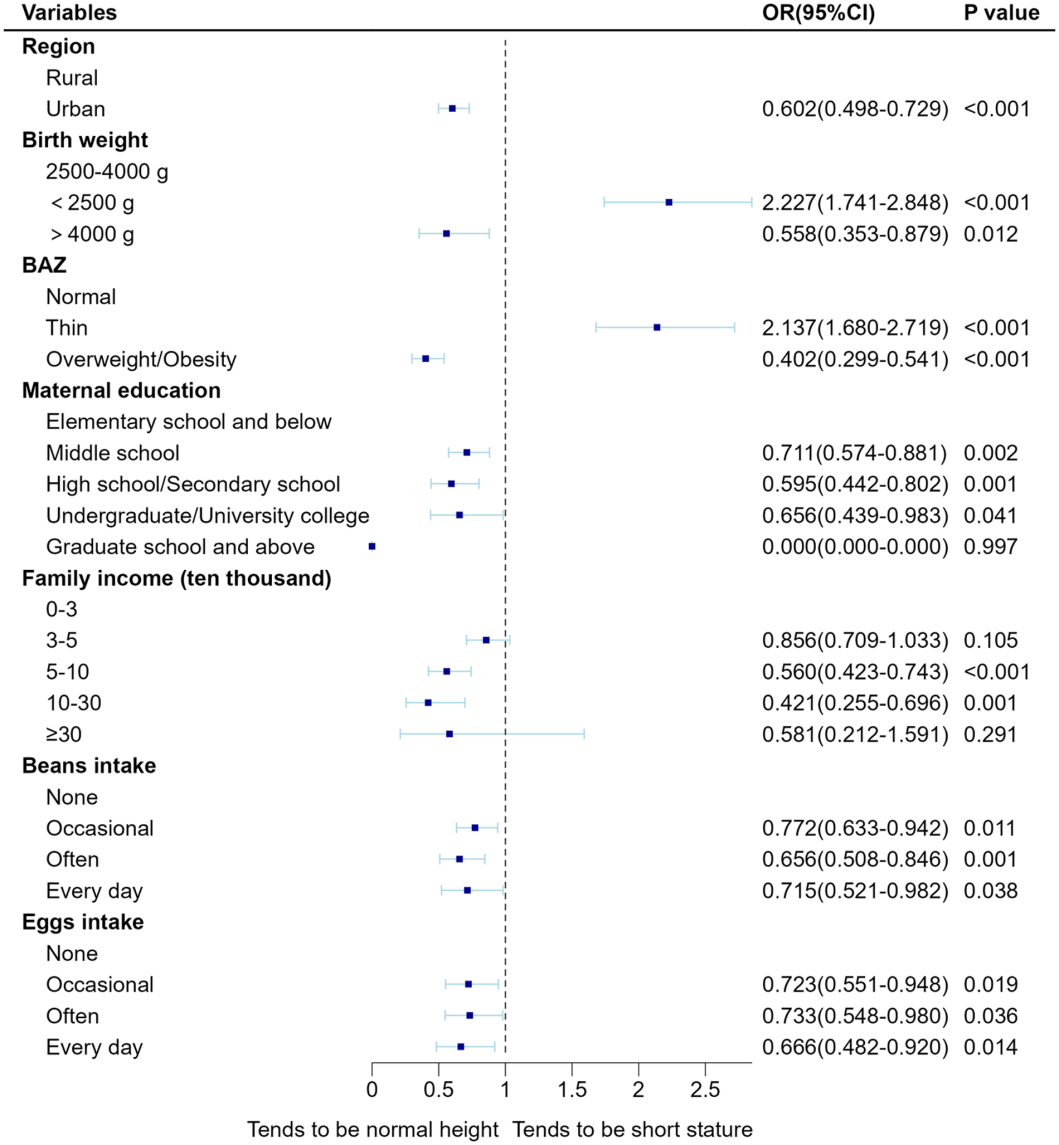
Fully adjusted forest plot of multivariate logistic regression of short stature in children.

**Figure 3:**
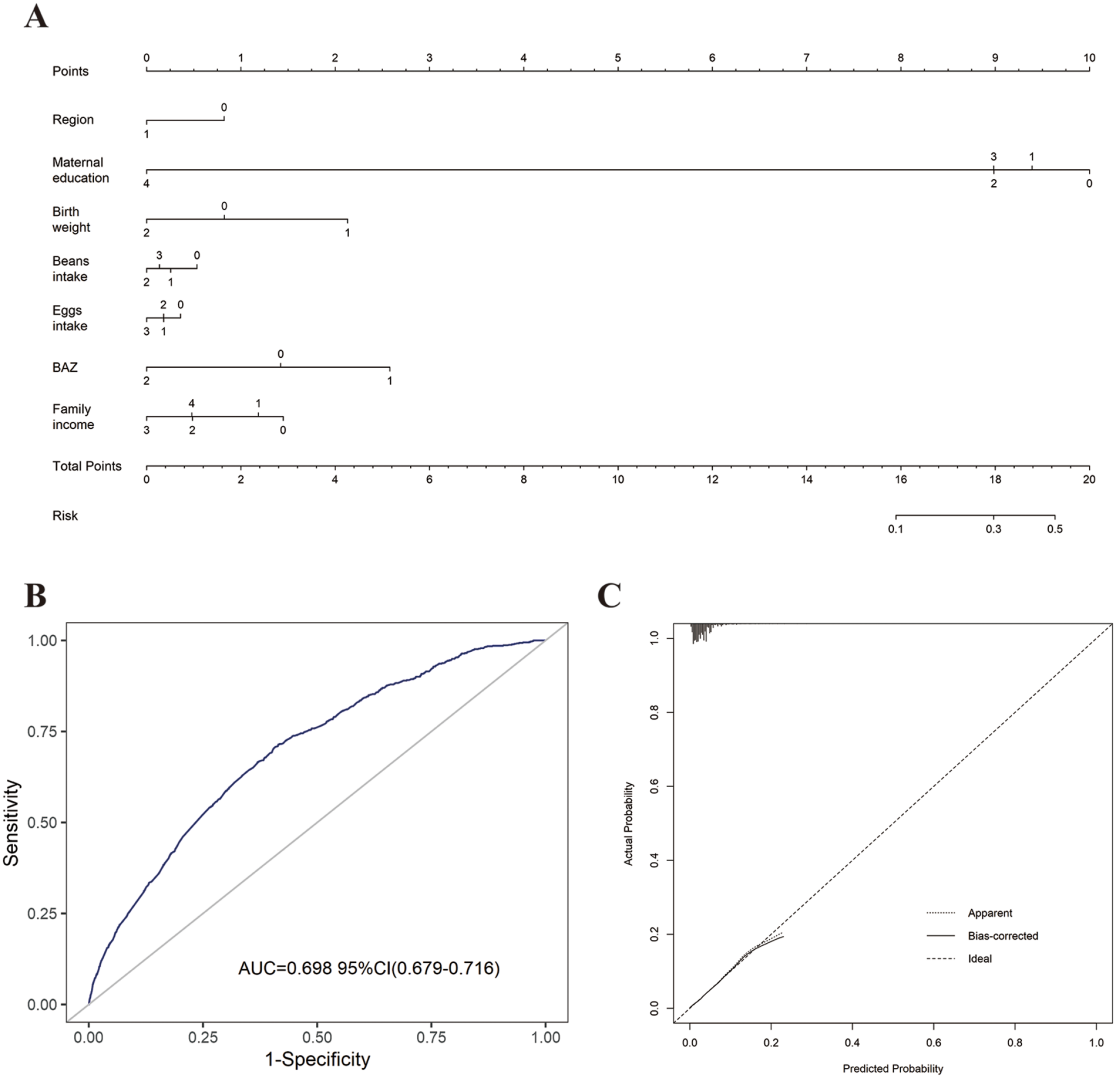
Predictive nomogram model **(A)** for shortness in children aged 3–18 years and evaluation of model. **(B)** Area under the ROC of the nomogram model; **(C)** the calibration curve of the nomogram model. Dashed line represents ideal prediction, and solid line represents observed nomogram. [Assignment of variables in the model: Regions, Rural = 0, Urban = 1; Maternal education, Elementary school and below = 0, Middle school = 1, High school/Secondary school = 2, Undergraduate/University college = 3, Graduate school and above = 4; Birth weight, 2,500 g–4,000 g = 0, <2,500 g = 1, ≥4,000 g = 2; Family income (RMB per year), 0–30,000 = 0, 30,000–50,000 = 1, 50,000–100,000 = 2,100,000–300,000 = 3, ≥300,000 = 4; Beans intake frequency, None = 0, Occasion = 1, Often = 2, Every day = 3; BAZ, BMI z score (BMI, body mass index), Normal = 0, Thin = 1, Overweight/Obesity = 2.).

As gender and age are immutable demographic variables, we adjusted them as confounders to obtain Model 2 (Supplementary Appendix S6). Next, other confounders were adjusted together to create Model 3 for examination (Supplementary Appendix S6). As shown in Figure 2, lower birth weight and thinness were significant risk factors for the development of short stature in children after multivariate adjustment (*P* < 0.001). Higher maternal education, higher annual family income, living in an urban area, and a higher frequency of bean and egg intake were negatively correlated with short stature (*P* < 0.001). Finally, a column-line graphical model of risk factors associated with short stature in children aged 3–18 years in Hainan Province was established based on the identified significant correlations (Figure 3). The area under the curve (AUC) of the receiver operating characteristic (ROC) curve was 0.698 (95% CI: 0.679–0.716), suggesting a fair degree of model fit and accuracy.

## 4 Discussion

In this large-scale cross-sectional study, we aimed to investigate the current status of short stature in children in Hainan Province and to identify nongenetic factors of short stature in children in Hainan Province. The standardised prevalence of short stature (with added weights) for children aged 3–18 years in Hainan Province was calculated as 3.01% by measuring the height of 26,189 children. Factors significantly associated with short stature included birth weight, BAZ, maternal education, annual family income, and frequency of bean intake (*P* < 0.001).

Several previous studies (34, 35) have clearly articulated the correlation between low birth weight and short stature in related areas. According to our findings, children born with low birth weight (<2,500 g) may be 2.5 times more likely to have short stature than children with normal birth weight (12.35% vs. 4.91%, *P* < 0.001). Therefore, the birth of low-birth-weight babies should be prevented as much as possible, and more attention should be given to the height growth of low-birth-weight babies. In addition, the effects of children's body mass index (BMI) on height reported in previous studies have been inconsistent. Therefore, we also included children's BAZ in this study. We found that thinness was positively associated with the incidence of short stature and that overweight/obesity was negatively correlated with short stature. A study in Israel in 2019 reported that excessive BMI did not prevent height growth compared with a normal BMI (36). However, some studies (9, 37) have shown that BMI is negatively correlated with height growth. There are discrepancies among the findings of different studies, perhaps due to the various populations of studies and statistical methods. These contradictory results indicate that the relationship between BMI and height growth is not clearly defined, and further research is needed. However, we believe that maintaining a normal BMI may be more beneficial to children's growth and development. Located at the southern tip of China and on the northern edge of the tropics, Hainan Province still lags significantly behind other provinces in China in terms of economic development. Furthermore, the level of urbanization across the province is also below the national average (38). According to our research, the prevalence of short stature in Hainan Province is higher than that among children in China, which stands at 2.4% (7). In some developed countries in Europe, the prevalence rate is relatively low. A research study indicates that the prevalence of short stature among children in the England region of Europe is approximately 1.82% (2). In Spain, a 2019 study showed that the detection rate of short stature among children was less than 1% (39). However, in some developing countries in Asia, the prevalence of stunting in children is relatively high. For instance, the prevalence of short stature among children in Saudi Arabia was 15% in 2015 (5). In Jordan, the prevalence among children was approximately 4.9% in 2016 (6). Across five South Asian countries (including Bangladesh, India, Nepal, Maldives, and Pakistan), the prevalence of stunting among children is 38% (40). Specifically, our findings show that the prevalence of stunted growth among children in Hainan Province (3.01%) is higher than in these developed countries but lower than in these developing countries. At the same time, these data also indicate that the prevalence is lower in developed countries than in developing countries. In 2010, it was estimated that 171 million children had stunting, of whom 167 million were in developing countries (41). Economic differences exist not only between countries and areas but also between rural and urban areas in the same area. In our study, the prevalence in rural children was more than twice that in urban children (3.76% vs. 1.71%), which was also statistically significant according to the logistic regression analysis model. The study conducted by Jia Ma et al. on the spatial and demographic differences in short stature among school-aged children also reached the same conclusion (32). Moreover, it is a common issue in most low- and middle-income countries (6, 42). In 2017, a study conducted by Liu Sisi et al. found that the prevalence of short stature among children aged 6–16 years in the Jining area of Shandong Province, China, was 3.15% (10). In 2015, the prevalence of short stature among children aged 7–18 years in Anhui Province was 3.16% (43). However, due to differences in the timing of the surveys and the diagnostic criteria used, we cannot simply assume that the prevalence rate of short stature among children in Hainan Province is the same as in other provinces of China.

We found that among the family-related factors, maternal education and family income were strongly correlated with short stature in children. This finding is consistent with the results of several previous studies that have shown a positive correlation among parental education, family income and children's height (34, 44–46); this could be because in some lower socioeconomic status households, compared with higher socioeconomic status households, there is a poorer provision of diets for child growth, a greater chance of child infections, and less concern for child height. Children may be fed more nutritious food by parents with higher levels of education (44). Thus, genetic variation in height may be influenced by the socioeconomic status of the family, and this hypothesis may be tested more precisely in future studies. In addition to economic and family-related factors, nutrition is an important factor that affects children's height, and studies in Saudi Arabia have confirmed that malnutrition is a major risk factor for short stature in children (31). Beans and eggs are rich in protein, fibre, minerals and vitamins (47, 48). The diets of the children in our study were analysed, and a statistically significant association was found between the frequency of intake of beans and eggs and occurrence of short stature; we speculate that this may be because children with a low frequency of them intake may be somewhat malnourished or have nutritional imbalances that increase the probability of short stature occurring. No statistically significant associations were found between the frequency of intake of meat, fruits, staple foods, puffed snacks, fried snacks, drinks, biscuits/cake snacks and sweets/chocolate snacks and short stature in our study. Because height growth is a complex process, we believe that a variable dietary intake is more favourable for children's growth and development. This was a cross-sectional study, and more precise studies will be needed in the future to explore the relationship between short stature and dietary habits.

Finally, we constructed a predictive nomogram model. A predictive model with multiple significant factors can not only help to estimate the probability of short stature in children, but can also provide targeted guidance for the prevention and control of short stature based on the identified risk factors.

## 5 Conclusions

In summary, in this study, we assessed the prevalence of short stature among children in Hainan Province for the first time by measuring the height of 26,189 children aged 3–18 years. By comprehensively analysing the survey data of 26,189 children, we observed that short stature was mainly associated with area, birth weight, BAZ, maternal education, annual family income, and frequency of bean and eggs intake.

## 6 Strengths and limitations

The strengths of this study include the first large-scale survey of children and adolescent height in Hainan Province, which covered the whole province with a large and representative sample size. Second, through the adjustment of different models, nongenetically related factors markedly correlated with short stature were identified, and a risk prediction nomogram model was constructed. However, some potential limitations should be recognised. First, this was a cross-sectional study, precluding further comments on causality. Second, there is a lack of data for children aged 0–3 years, which is not representative of the prevalence throughout childhood.

## Data Availability

The original contributions presented in the study are included in the article/Supplementary Material, further inquiries can be directed to the corresponding author.
