# Supplementary material for: Prevalence and predictors of short stature in children aged 3–18 years in Hainan Province, China: a cross-sectional study
Source: Front Pediatr. 2025 Jan 20;13:1522060. doi: 10.3389/fped.2025.1522060 (PMC11788376; doi:10.3389/fped.2025.1522060)
Supplement: Supplementary file 1 [file Supplementaryfile1.docx]

Supplementary Material

# Supplemental Appendix 1

The specific sampling method is as follows. According to the administrative hierarchy, the two prefecture-level cities of Haikou and Sanya were stratified by district town/street, with all districts selected. Except for Haikou and Sanya, stratification was performed according to the order of city/county-town/street. Towns/streets are the primary sampling units. The town/street level was stratified by kindergarten, primary school, middle school, and high school. All schools served as secondary sampling units. Schools were randomly selected in the sampled townships or streets stratified by kindergarten, elementary school, middle school, and high school. The sampled schools as a whole and all students participated in this survey. If the sampled township or street did not have a school of the appropriate tier, the school of the appropriate tier was selected from the closest neighbouring district.

In this study, we calculated the weights for the cross-sectional samples. The calculation steps are as follows: 1) We assume that the probability of selecting a male or female from any age group in any city or county is equal to the ratio of the number of individuals surveyed in that category to the total population of that category in Hainan. Therefore, the weight is the reciprocal of this ratio. For example, if the total number of 0-year-old male children in Haikou in 2020 is 13,125 and the sample size of 0-year-old male children in Haikou in this cross-sectional survey is 1,114, then the weight for 0-year-old male children in Haikou in this survey is 13,125 / 1,114 = 11.78. 2) Following this method, we calculated the weights for each cell. 3) In the statistical description of this cross-sectional survey, each study subject was weighted before further statistical analysis was conducted.

# Supplemental Appendix 2

Method of height measurement

Measurements were taken while the subject wore only a vest and shorts (shoes, socks, hat and outer clothing removed). The subject stood on the pedals in an upright position, chest out, abdomen in, arms hanging down naturally, heels closed, feet angled at approximately 60 degrees, knees together and straight. The subject looked straight ahead, with the upper edge of the auricle and the lower edge of the eye socket on both sides at the same level. The heels, hips and middle of the corners of both shoulder blades touched the riser simultaneously, and the head remained in a square position. The measurer observed the subject's posture and confirmed that the posture was correct before taking measurements. The measurer's eyes were at the same level as the bottom surface of the slide, and the value shown on the column corresponding to the bottom surface of the slide was read in “centimetres” accurate to 0.1 cm.

Method of weight measurement

The measuring instrument was a vertical electronic weighing scale (seca255) with a maximum weight of 200 kg and a divisional value of 100 g. The scale was placed in a stable position, the zero point was calibrated, and in case of deviation, the zero point was calibrated in accordance with the instructions for use of the "electronic weighing scale". Before measurement, the subject urinated and defecated; removed shoes, socks, hats and outerwear; and wore only a vest and shorts. The measurement was started only when the electronic screen read 0.00. The child stood barefoot in the centre of the scale pedal and kept his/her body steady, without shaking or touching other objects. The tester recorded the reading to two decimal places when the display remained stable.

# Supplemental Appendix 3

Urban are defined as provincial capitals, locations of prefecture-level cities, county towns, and locations of main urban areas at the county level. Rural areas are defined as villages, including townships, natural villages, and suburbs, in cities and counties (excluding the main urban areas of foreign counties). Children in urban areas are those who have been living in urban areas of the province for a long time or who have moved in from abroad but have lived in the main urban areas of the province for a period of time longer than two-thirds of their age. Children in rural areas are those who have been living in townships for a long time and are registered in townships or who have moved in from abroad but have lived in the townships for a period of time longer than two-thirds of their age.

# Supplemental Appendix 4:

Variable assignment table.

| Variables | The assignment |
| --- | --- |
| Gender | Girl = 0, Boy = 1 |
| Regions | Rural = 0, Urban = 1 |
| Parental education | Elementary school and below = 0, Middle school = 1, High school/Secondary school = 2, Undergraduate/University college = 3, Graduate school and above = 4 |
| Gestational age (weeks) | 37-42 = 0, ＜37 = 1, ≥42 = 2 |
| Birth weight | 2500 g-4000 g = 0, ＜2500 g = 1, ≥4000 g = 2 |
| BAZ | Normal = 0, Thin = 1, Overweight/Obesity = 2 |
| Family income (ten thousand) | 0-30000 = 0, 30000-50000 = 1, 50000-100000= 2, 100000-300000=3, ≥300000 = 4 |
| Beans / Eggs / Meats / Fruits / Staple intake | None = 0，Occasion = 1，Often = 2，Every day = 3 |
| Puffed snack / Fried snack / Biscuits-Cakes / Drinks / Sweets-Chocolate | None = 0，Occasion = 1，Often = 2，Every day = 3 |
| Texture of staple foods | Gruel = 0, Thick porridge = 1, Thin rice = 2, Dry rice = 3 |
| Outdoor activities / Electronic screens | ＜1 h = 0, 1-2 h = 1, >2 h = 2 |
| Short Stature | No = 0, Yes = 1 |

# Supplemental Appendix 5:

| Variables | Children with short stature (n=753) | Non-diseased (n=25,436) | χ2 / t | P value |
| --- | --- | --- | --- | --- |
| Lifestyle-related factors |  |  |  |  |
| Texture of staple foods (%) |  |  | 30.779 | ＜0.001 |
| Gruel | 335 (44.49) | 9449 (37.15) |  |  |
| Thick porridge | 83 (11.02) | 2843 (11.18) |  |  |
| Thin rice | 123 (16.34) | 3576 (14.06) |  |  |
| Dry rice | 212 (28.15) | 9568 (37.62) |  |  |
| Puffed snack intake (%) |  |  | 7.478 | 0.058 |
| None | 133 (17.66) | 4159 (16.35) |  |  |
| Occasional | 547 (72.64) | 1,9344 (76.05) |  |  |
| Often | 66 (8.77) | 1808 (7.11) |  |  |
| Every day | 7 (0.93) | 125 (0.49) |  |  |
| Fried snack intake (%) |  |  | 2.770 | 0.428 |
| None | 90 (11.95) | 2599 (10.22) |  |  |
| Occasional | 620 (82.34) | 2,1383 (84.06) |  |  |
| Often | 42 (5.58) | 1393 (5.48) |  |  |
| Every day | 1 (0.13) | 61 (0.24) |  |  |
| Biscuits-Cakes intake (%) |  |  | 3.164 | 0.367 |
| None | 30 (3.99) | 992 (3.90) |  |  |
| Occasional | 596(79.15) | 1,9562 (76.91) |  |  |
| Often | 122 (16.20) | 4749 (18.67) |  |  |
| Every day | 5 (0.66) | 133 (0.52) |  |  |
| Drinks intake (%) |  |  | 2.737 | 0.434 |
| None | 49 (6.51) | 1665 (6.55) |  |  |
| Occasional | 602 (79.95) | 2,0268 (79.68) |  |  |
| Often | 94 (12.48) | 3349 (13.17) |  |  |
| Every day | 8 (1.06) | 154 (0.60) |  |  |
| Sweets-Chocolate intake (%) |  |  | 3.800 | 0.284 |
| None | 78 (10.36) | 2519 (9.90) |  |  |
| Occasional | 596 (79.15) | 2,0696 (81.36) |  |  |
| Often | 77 (10.23) | 2128 (8.37) |  |  |
| Every day | 2 (0.26) | 93 (0.37) |  |  |
| Electronic screens(day) (%) |  |  | 8.096 | 0.017 |
| ＜1 h | 428 (56.84) | 1,5369 (60.42) |  |  |
| 1-2 h | 173 (22.97) | 5907 (23.22) |  |  |
| >2 h | 152 (20.19) | 4160 (16.35) |  |  |
| Outdoor activities(day) (%) |  |  | 4.060 | 0.131 |
| ＜1 h | 251 (33.33) | 9319 (36.64) |  |  |
| 1-2 h | 264 (35.06) | 8755 (34.42) |  |  |
| >2 h | 238 (31.61) | 7362 (28.94) |  |  |

BMI z: BMI z: BMI z score. The data are expressed as medians (interquartile ranges) or counts (percentages). The P value was calculated using t test or chi-squared test. A p value < 0.05 indicated statistical significance. Weekly intake frequency was classified as none, often (3-5 times), occasional (1-2 times) or daily.

# Supplemental Appendix 6:

Identification of significant contributing factors for short stature using univariate and multivariable logistic regression analyses in 3- to 18-year-old children

| Factors | | | | Short stature | | | | | | | | | | | |
| --- | --- | --- | --- | --- | --- | --- | --- | --- | --- | --- | --- | --- | --- | --- | --- |
|  |  |  |  | OR | | | | 95% CI | | | | P | | | |
| **Unadjusted** | | | |  | | | |  | | | |  | | | |
| Area | | | |  | | | |  | | | |  | | | |
| Rural | | | | Reference | | | | | | | | | | | |
| Urban | | | | 0.435 | | | | 0.363-0.521 | | | | <0.001 | | | |
| Birth weight | | | |  | | | |  | | | |  | | | |
| 2500-4000 g | | | | Reference | | | |  | | | |  | | | |
| <2500 g | | | | 2.672 | | | | 2.134-2.961 | | | | <0.001 | | | |
| >4000 g | | | | 0.578 | | | | 0. 369-0.906 | | | | 0.017 | | | |
| BAZ | | | |  | | | |  | | | |  | | | |
| Normal | | | | Reference | | | |  | | | |  | | | |
| Thin | | | | 2.359 | | | | 1.864-2.985 | | | | <0.001 | | | |
| Overweight/Obesity | | | | 0.349 | | | | 0.260-0.468 | | | | <0.001 | | | |
| Maternal education | | | |  | | | |  | | | |  | | | |
| Elementary school and below | | | | Reference | | | |  | | | |  | | | |
| Middle school | | | | 0.596 | | | | 0.490-0.723 | | | | <0.001 | | | |
| High school/Secondary school | | | | 0.356 | | | | 0.274-0.463 | | | | <0.001 | | | |
| Undergraduate/University College | | | | 0.263 | | | | 0.191-0.361 | | | | <0.001 | | | |
| Graduate school and above | | | | 0.000 | | | | 0.000-- | | | | 0.997 | | | |
| Family income (RMB per year) (%) | | | |  | | | |  | | | |  | | | |
| 0-30000 | | | | Reference | | | |  | | | |  | | | |
| 30000-50000 | | | | 0.696 | | | | 0.580-0.835 | | | | <0.001 | | | |
| 50000-100000 | | | | 0.367 | | | | 0.282-0.478 | | | | <0.001 | | | |
| 100000-300000 | | | | 0.219 | | | | 0.137-0.351 | | | | <0.001 | | | |
| ≥300000 | | | | 0.288 | | | | 0.107-0.775 | | | | 0.014 | | | |
| Beans intake | | | |  | | | |  | | | |  | | | |
| None | | | | Reference | | | |  | | | |  | | | |
| Occasional | | | | 0.661 | | | | 0.560-0.781 | | | | <0.001 | | | |
| Often | | | | 0.570 | | | | 0.455-0.714 | | | | <0.001 | | | |
| Every day | | | | 0.590 | | | | 0.447-0.780 | | | | <0.001 | | | |
| Eggs intake | | | |  | | | |  | | | |  | | | |
| None | | | | Reference | | | |  | | | |  | | | |
| Occasional | | | | 0.671 | | | | 0.552-0.817 | | | | <0.001 | | | |
| Often | | | | 0.603 | | | | 0.487-0.748 | | | | <0.001 | | | |
| Every day | | | | 0.459 | | | | 0.360-0.585 | | | | <0.001 | | | |
| **Age-, and gender-adjusted** | | | |  | | | |  | | | |  | | | |
| Area | | | |  | | | |  | | | |  | | | |
| Rural | | | | Reference | | | |  | | | |  | | | |
| Urban | | | | 0.441 | | | | 0.368-0.529 | | | | <0.001 | | | |
| Birth weight | | | |  | | | |  | | | |  | | | |
| 2500-4000 g | | | | Reference | | | |  | | | |  | | | |
| <2500 g | | | | 2.685 | | | | 2.143-3.363 | | | | <0.001 | | | |
| >4000 g | | | | 0.547 | | | | 0.349-0.859 | | | | 0.009 | | | |
| BAZ | | | |  | | | |  | | | |  | | | |
| Normal | | | | Reference | | | |  | | | |  | | | |
| Thin | | | | 2.324 | | | | 1.836-2.942 | | | | <0.001 | | | |
| Overweight/Obesity | | | | 0.342 | | | | 0.255-0.459 | | | | <0.001 | | | |
| Maternal education | | | |  | | | |  | | | |  | | | |
| Elementary school and below | | | | Reference | | | |  | | | |  | | | |
| Middle school | | | | 0.599 | | | | 0.491-0.730 | | | | <0.001 | | | |
| High school/Secondary school | | | | 0.358 | | | | 0.274-0.468 | | | | <0.001 | | | |
| Undergraduate/University college | | | | 0.265 | | | | 0.191-0.368 | | | | <0.001 | | | |
| Graduate school and above | | | | 0.000 | | | | 0.000-- | | | | 0.997 | | | |
| Family income (RMB per year) (%) | | | |  | | | |  | | | |  | | | |
| 0-30000 | | | | Reference | | | |  | | | |  | | | |
| 30000-50000 | | | | 0.699 | | | | 0.582-0.839 | | | | <0.001 | | | |
| 50000-100000 | | | | 0.372 | | | | 0.285-0.484 | | | | <0.001 | | | |
| 100000-300000 | | | | 0.224 | | | | 0.140-0.360 | | | | <0.001 | | | |
| ≥300000 | | | | 0.292 | | | | 0.109-0.785 | | | | 0.015 | | | |
| Beans intake | | | |  | | | |  | | | |  | | | |
| None | | | |  | | | |  | | | |  | | | |
| Occasional | | | | 0.662 | | | | 0.560-0.781 | | | | <0.001 | | | |
| Often | | | | 0.569 | | | | 0.455-0.713 | | | | <0.001 | | | |
| Every day | | | | 0.584 | | | | 0.442-0.772 | | | | <0.001 | | | |
| Eggs intake | | | |  | | | |  | | | |  | | | |
| None | | | | Reference | | | |  | | | |  | | | |
| Occasional | | | | 0.861 | | | | 0.673-1.100 | | | | 0.231 | | | |
| Often | | | | 0.756 | | | | 0.588-0.972 | | | | 0.029 | | | |
| Every day | | | | 0.631 | | | | 0.507-0.786 | | | | <0.001 | | | |
| **Multivariable adjusted** | | | |  | | | |  | | | |  | | | |
| Area | | | |  | | | |  | | | |  | | | |
| Rural | | | | Reference | | | |  | | | |  | | | |
| Urban | | | | 0.602 | | | | 0.498-0.729 | | | | <0.001 | | | |
| Birth weight | | | |  | | | |  | | | |  | | | |
| 2500-4000 g | | | | Reference | | | |  | | | |  | | | |
| <2500 g | | | | 2.227 | | | | 1.741-2.847 | | | | <0.001 | | | |
| >4000 g | | | | 0.558 | | | | 0.353-0.879 | | | | 0.012 | | | |
| BAZ | | | |  | | | |  | | | |  | | | |
| Normal | | | | Reference | | | |  | | | |  | | | |
| Thin | | | | 2.137 | | | | 1.680-2.719 | | | | <0.001 | | | |
| Overweight / Obesity | | | | 0.402 | | | | 0.299-0.541 | | | | <0.001 | | | |
| Maternal education | | | |  | | | |  | | | |  | | | |
| Elementary school and below | | | | Reference | | | |  | | | |  | | | |
| Middle school | | | | 0.711 | | | | 0.575-0.881 | | | | 0.002 | | | |
| High school / Secondary school | | | | 0.595 | | | | 0.442-0.802 | | | | 0.001 | | | |
| Undergraduate / University college | | | | 0.657 | | | | 0.439-0.982 | | | | 0.041 | | | |
| Graduate school and above | | | | 0.000 | | | | 0.000-- | | | | 0.997 | | | |
| Family income (RMB per year) (%) | | | |  | | | |  | | | |  | | | |
| 0-30000 | | | | Reference | | | |  | | | |  | | | |
| 30000-50000 | | | | 0.856 | | | | 0.709-0.709 | | | | 0.105 | | | |
| 50000-100000 | | | | 0.560 | | | | 0.423-0.423 | | | | <0.001 | | | |
| 100000-300000 | | | | 0.421 | | | | 0.255-0.696 | | | | 0.001 | | | |
| ≥300000 | | | | 0.581 | | | | 0.212-1.591 | | | | 0.291 | | | |
| Beans intake | | | |  | | | |  | | | |  | | | |
| None | | | | Reference | | | |  | | | |  | | | |
| Occasional | | | | 0.772 | | | | 0.633-0.942 | | | | 0.011 | | | |
| Often | | | | 0.656 | | | | 0.508-0.846 | | | | 0.001 | | | |
| Every day | | | | 0.715 | | | | 0.521-0.982 | | | | 0.039 | | | |
| Eggs intake | | | |  | | | |  | | | |  | | | |
| None | | | | Reference | | | |  | | | |  | | | |
| Occasional | | | | 0.723 | | | | 0.551-0.948 | | | | 0.019 | | | |
| Often | | | | 0.733 | | | | 0.548-0.980 | | | | 0.036 | | | |
| Every day | | | | 0.666 | | | | 0.482-0.920 | | | | 0.014 | | | |

Abbreviations: OR, odds ratio; 95% CI, 95% confidence interval; BAZ, BMI z score (BMI, body mass index). A p value < 0.05 indicated statistical significance. The multivariable-adjusted variables included age, gender, electronic screen time, time spent engaging in outdoor activities, gestational age, staple food intake, fruit intake, meat intake, puffed snack intake, fried snack intake, cake/biscuit snack intake, drink intake, and sweet/chocolate snack intake.

**
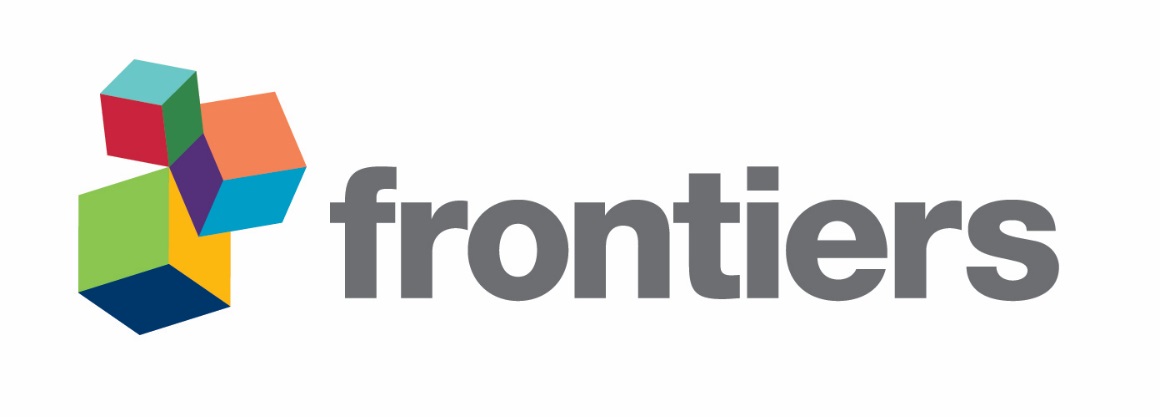
**
